# A systematic review of decision tools for process selection and performance improvement in manufacturing

**DOI:** 10.1007/s00170-025-16806-y

**Published:** 2025-10-21

**Authors:** Ziyad Sherif, Konstantinos Salonitis

**Affiliations:** Sustainable Manufacturing Systems Centre, Faculty of Engineering and Applied Sciences, Cranfield University, Cranfield, MK43 0AL UK

**Keywords:** Comparative analysis, Decision-making, Manufacturing processes

## Abstract

The growing complexity of manufacturing processes and the increasing diversity of decision-making tools present challenges in selecting effective approaches for process optimisation. Many existing tools are either too narrowly focused or inconsistently applied across sectors, limiting their broader impact. Additionally, the lack of clear integration strategies often hinders their full implementation in industrial settings. This systematic review examines decision-making tools that enable comparative assessments applied at the unit process level in manufacturing, covering both the selection between competing manufacturing routes and the optimisation of specific processes. A total of 37 journal articles were selected through a structured database search and evaluation process. The review analyses commonly used tools such as Multi-Criteria Decision Analysis (MCDA), Life Cycle Assessment (LCA), and Direct Comparison, highlighting their applications, benefits and limitations. Findings show that MCDA offers robust, multi-dimensional evaluations but is often constrained by complexity and data demands. In contrast, simpler methods like Direct Comparison provide more accessible insights but with a limited scope. Advanced tools such as Deep Learning and Computational Simulations hold promise but face challenges in scaling beyond the process level. Notably, there is limited integration of sustainability metrics within process-level decision-making. To address this, the study proposes a structured framework to guide future research and implementation, focusing on data management, AI integration and tool scalability. The results highlight the need for hybrid approaches that combine different tools to balance trade-offs and support long-term sustainability and operational efficiency in manufacturing systems.

## Introduction

Manufacturing is a cornerstone of modern economies, driving technological advancements, economic growth and societal development. At its core, manufacturing encompasses a plethora of processes, systems and strategies that collectively contribute to producing goods and services. It is responsible for US$35tn of output in 2024 globally with 5.46 m enterprises [1]. The complexity of manufacturing systems, comprising products, equipment, personnel, information and control mechanisms, necessitates robust tools and methods to ensure efficiency, sustainability and competitiveness in the global market [2, 3].

The importance of manufacturing processes cannot be overstated, as they form the foundation of production systems, influencing product quality, cost-efficiency and environmental impact. Manufacturing processes are not just isolated operations but integral components of larger systems that must be optimised to meet market demands and regulatory standards. For example, machining a metal component for an automotive engine involves precision cutting and shaping to achieve the required tolerances and surface finish. This single-unit process directly impacts the engine's performance and longevity, as well as the overall manufacturing cost and environmental footprint. At the same time, the adoption of advanced 4.0 technology, such as artificial intelligence, robotics and the Internet of Things (IoT), has the potential to improve overall efficiency and productivity while reducing costs. However, leveraging these technologies adds complexity to operating and optimising such processes [4].

Therefore, decision-making at the unit process level, such as selecting between machining or casting for a specific component, plays a critical role in determining the overall success of manufacturing systems. Given the multifaceted nature of manufacturing, decision-makers are often confronted with complex challenges that require careful consideration of multiple factors. These factors include cost, quality and sustainability [5]. While heuristics and rules of thumb are often used for decision-making in manufacturing due to their simplicity and ease of application, they fall short in addressing the growing complexity of modern manufacturing systems. These methods lack the ability to systematically analyse trade-offs between conflicting factors and are prone to subjective biases. In contrast, structured decision-making tools provide a robust, data-driven approach, enabling manufacturers to handle the intricate interdependencies of their operations and optimise processes with greater accuracy and scalability. To navigate these challenges, various decision-making tools and approaches have been developed. These tools are established through the support of a variety of techniques ranging from benchmarking and monitoring methods [6, 7] to multi-criteria decision analysis (MCDA) [8, 9] and the increasingly widespread use of artificial intelligence (AI) [10, 11], each offering unique advantages and limitations depending on the context of their application.

Decision-making tools are particularly vital at the process level, where detailed analysis of specific manufacturing processes can significantly improve efficiency and sustainability [12]. Furthermore, comparing processes is paramount, enabling a more informed evaluation of different methods, practices and standards. Comparative analysis allows for a nuanced understanding of how various processes perform under similar or differing conditions, making it possible to identify the most suitable possibility for given requirements [13–15]. This analysis also serves as the foundation for performance evaluation by establishing benchmarks and competitive priorities [16].

Such decision-making is especially important in manufacturing, where decisions often need to be made regarding which processes to implement or enhance based on multiple, and sometimes conflicting, factors. This study systematically reviews decision-making concepts and approaches targeting unit manufacturing processes. The review aims to explore the tools available for process-level decision-making, including those with broader implications for system-level decisions. The primary focus is on methods involving comparative evaluation. By doing so, this study seeks to uncover strategies that evaluate individual processes and provide a broader understanding of how these processes perform relative to one another. The scope of this review is intentionally broad, encompassing diverse industries and manufacturing contexts, to identify common trends, gaps in the literature and opportunities for further research.

The findings of this study can guide manufacturers, researchers and policymakers in selecting and implementing effective decision-making tools to optimise production processes. By providing a comprehensive overview of comparative methods, the study also highlights areas for innovation and encourages the development of tailored tools for specific manufacturing needs.

## Literature review

### Decision-making tools in manufacturing

Decision-making tools are crucial for optimising and improving manufacturing processes. These tools offer structured methods for evaluating multiple factors, such as cost, quality, sustainability, and efficiency, which are essential for driving improvements in manufacturing operations [17, 18]. Over time, various approaches have been developed to aid decision-makers in balancing trade-offs and selecting the best strategies in complex industrial settings.

These tools provide a means of systematically assessing processes and making informed decisions across different operational levels, from individual production processes to broader system-wide evaluations. Depending on the operational focus, the impacts of these tools can vary from direct improvements in production at the process level [19] to more comprehensive gains in sustainability or resource optimisation when applied at the system or organisational level [20]. Their procedure may range from qualitative methods to more data-driven, quantitative models, helping manufacturers assess both current operations and future scenarios. By applying decision-making tools at different levels of manufacturing, companies can address specific operational challenges while improving overall production efficiency and strategic alignment [21, 22]. The adoption and integration of such tools have led to notable advancements in numerous operating aspects across various industries [23, 24]. However, challenges remain, particularly in ensuring data availability, managing complexity, and adapting tools to new technologies and manufacturing contexts [25, 26].

### Analysis of manufacturing processes

Analysing manufacturing processes is essential for identifying inefficiencies, optimising resource use and improving overall production quality. This analysis often includes evaluating different processing levels, from individual processes to entire production systems. The focus can be exclusively on particular processes. Still, it can also adopt a bottom-up approach where improvements at the process level trickle upwards, ultimately impacting the entire production system or facility [27]. Various methodologies are employed to achieve these goals, each focusing on different aspects of the manufacturing process [28].

The need for thorough and systematic process evaluation has driven the development of more advanced methods for capturing the complexity of modern manufacturing systems. As new challenges arise, such as shifts toward more sustainable or digital manufacturing practices, further research and innovation in process analysis will be needed to address these evolving requirements [29, 30].

### Previous related work

Several review studies have explored various decision-making tools and methods within different manufacturing contexts, offering insights into their applications, benefits and limitations. For instance, Ng et al. [31] focused on the application of machine learning (ML) tools in additive manufacturing, particularly in areas such as quality control, process optimisation and material formulation. This review provided a comprehensive analysis of how machine learning enhances these aspects within the additive manufacturing sector, highlighting the strengths and the areas where further research is needed. In parallel, a study by Mallioris et al. [32] on predictive maintenance applications across diverse manufacturing sectors discovered a high dependence on ML approaches to predictive maintenance across various sectors.

In a more expansive context, several reviews have explored the application of AI in manufacturing. Elahi et al. [33] examined the implementation of AI techniques throughout the lifecycle of industrial equipment, from design to recycling, emphasising the role of AI in optimising process control and decision-making across the major phases of an industrial equipment lifecycle. Similarly, Hoffmann and Reich [34] specifically assessed the use of AI in visual quality control for defect detection, identifying gaps in current research, particularly the limited use of AI in explaining decision-making processes in quality assurance.

Other reviews concentrated on the various selection and optimisation techniques included under the umbrella of MCDA tools. For instance, a study by Raja and Rajan [35] explored utilising MCDA modelling in additive manufacturing, mainly focusing on polymer technology. They explored the challenges and opportunities presented by these methods, underscoring their importance in overcoming specific optimisation issues. Similarly, a review by Jamwal et al. [36] examined the use of MCDA techniques in sustainable manufacturing, offering insights into how these tools support decision-making processes to achieve sustainability goals within various manufacturing sectors. The application ranged from material selection to scheduling to identifying factors and drivers of sustainable manufacturing.

Broad reviews have also been conducted on target monitoring approaches. For instance, Ravelomanantsoa et al. [37] explored approaches devoted to the design and implementation of performance measurement systems for production functions spanning supply, manufacturing, and distribution. They aspired to aid companies and consultants in choosing the most appropriate approach based on the availability and depth of performance indicators.

Lastly, studies have focused on integrating advanced technologies in manufacturing decision-making. For instance, Inturi et al. [38] review of digital twins in transmission and industrial machinery provided a detailed analysis of their definitions, applications, and performance in enhancing manufacturing and lifecycle management. Similarly, Wu and Liang [39] investigated intelligent data acquisition and presentation methods in manufacturing, emphasising the integration of process knowledge, management, and decision-making to enhance operational efficiency. Likewise, Sekaran et al. [40] explored the application of virtual reality (VR) in digital factory simulations. These illustrate how VR aids in the efficient operation of manufacturing facilities. These studies have provided unique insights into integrating these technologies into manufacturing decision-making.

Table 1 compares surveys according to whether they are sector-specific or general across industries, whether they focus on a particular decision-making tool or cover multiple tools, and whether their analysis is framed at the process level rather than only at broader product, system, or facility scales.

**Table 1:** Comparison of the scope of previous surveys and the present study

Ref	Year	Non-sector specific	Non-tool specific	Process level
[31]	2024	x	x	✓
[32]	2024	✓	x	x
[33]	2023	x	x	x
[34]	2023	x	x	x
[35]	2023	x	x	✓
[36]	2020	✓	x	✓
[37]	2017	✓	x	x
[38]	2024	x	x	✓
[39]	2024	✓	x	x
[40]	2021	✓	x	✓
This study	2024	✓	✓	✓

### Identified research gaps

Although previous reviews have offered valuable insights into specific tools, techniques, and industries, several important gaps remain. Many studies are limited in scope, concentrating on a single sector or a narrow group of tools, which restricts opportunities for cross-sector learning at the unit-process level. The present review addresses this by synthesising studies from multiple sectors and tool families, allowing comparisons that reveal both commonalities and sector-specific practices. A further limitation is that much of the literature evaluates processes in isolation without incorporating a comparative perspective. This gap is addressed here through the inclusion of studies with a clear comparative element, as well as an analysis of how such comparisons are constructed and interpreted at the process level.

Previous reviews have also provided limited linkage between methods, impact areas, and bases of comparison, leaving uncertainty about which tools target particular outcomes or rely on specific indicators. In this review, these linkages are made explicit through systematic coding and cross-thematic analysis. Sustainability considerations are frequently treated separately from technical and economic factors, which reduces the ability to evaluate trade-offs. The integration of environmental, technical, and financial perspectives within this study highlights both areas of convergence and remaining gaps. Another shortcoming in earlier work has been the inconsistent treatment of operational levels, with little clarity on how process-level tools can scale to facility or system levels. This review classifies tools according to their operational scope and examines their scalability in greater detail.

Finally, practical advice is rarely provided in earlier reviews. In contrast, this paper offers a structured implementation guide with clear steps for adopting decision-making tools in manufacturing settings. By explicitly linking these research gaps with the contributions made, this review advances both academic understanding and practical application of decision-making tools at the manufacturing process level.

### Research questions

Based on the scope and focus of the review, the following research questions have been formulated:

1. What decision-making methods and approaches are currently used at the process level in manufacturing, and how do they vary in their application?
2. What are the key impacts and limitations of existing decision-making methods, and how can these be addressed in future research?
3. How can these methods be integrated into manufacturing systems to support effectiveness and scalability?

## Methodology

**Fig. 1:**
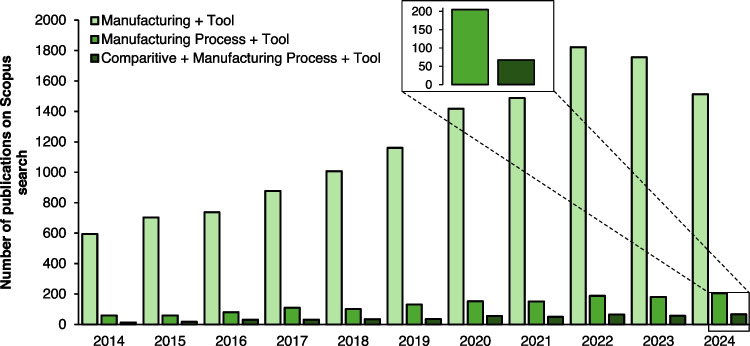
From 2014 to 2024, the number of articles retrieved from Scopus showed progressive keyword inclusion. It displays the keyword search results for 'Comparative Manufacturing Process Decision Tools', 'Manufacturing Process Decision Tools' and 'Manufacturing Decision Tools'

This study employed a systematic literature review (SLR) to provide a comprehensive overview of decision-making tools at the manufacturing process level. It focused on comparative analysis across or within different sectors. Figure 1 highlights the growing academic interest in decision-making tools for manufacturing. Over the past decade, the number of publications has increased steadily, reflecting a heightened focus on improving process efficiency and optimisation by utilising various tools and approaches.

Initial search queries using broad terms and synonyms like “manufacturing decision tools” demonstrated a general interest, yielding around 13,000 articles. However, when refining the search to focus on analyses between processes, the number of relevant publications narrowed to approximately 1,400 studies. Incorporating terms like “Comparative” has further reduced the results to 500 studies. This significant reduction points to the specialised nature of research in comparative decision-making at the manufacturing process level but also underscores its growing relevance within the academic community.

This trend not only signals an increasing recognition of the importance of comparative analysis in process decision-making but also indicates a shift toward more precise, data-driven evaluations. The analysis revealed that over time, research has increasingly emphasised the importance of comparing different manufacturing processes across and within sectors to identify optimal approaches for production effectiveness. These findings illustrate the broader transition within manufacturing from isolated process enhancement to holistic, comparative assessments aimed at fostering innovation and competitiveness.

Consequently, the systematic review method is ideal for synthesising and evaluating existing research. It allows for identifying knowledge gaps and provides robust insights that can inform future studies on decision-making tools for manufacturing processes. The PRISMA 2020 (Preferred Reporting Items for Systematic Reviews and Meta-Analyses) guidelines ensure transparency, accuracy, and comprehensiveness throughout the review process [41]. It offers a 27-item checklist and a flow diagram that guides this review's identification, screening, and inclusion phases, ensuring a clear and structured approach to reporting findings.

### Search strategy and databases

This review identified relevant literature using two primary databases: Scopus and Web of Science. These databases were selected because they cover a broad range of peer-reviewed manufacturing and decision-making research studies, making them highly suitable for this review. Keywords and Boolean operators such as "manufacturing process", “decision tools", and "comparative analysis" were combined to retrieve a comprehensive list of studies. Wildcards and synonyms were employed to capture variations of search terms, ensuring a broad yet focused set of results. The search strings used are displayed in Table 2.

**Table 2:** Literature review search strings

Database	String
Scopus	TITLE-ABS-KEY(("manufacturing process*") AND decision AND (tool OR approach OR model OR method OR analysis OR support) AND (compar* OR benchmark*))
Web of Science	TS = ("manufacturing process*" AND decision AND (tool OR approach OR model OR method OR analysis OR support) AND (compar* OR benchmark*))

The results were limited to publication dates (from 2010 onwards), peer-reviewed articles and studies written in English. This strategy aligned directly with the study’s objectives, enabling the identification of robust, process-level decision-making tools within manufacturing. By focusing on recent advancements and employing comparative analysis, the review ensures that established and emerging methods are captured, providing insights across multiple sectors.

To avoid reviewing duplicated papers, the entire lists of papers that appeared for each of the searching criteria in the two databases were exported to Zotero to be compared, and 347 duplicates were removed. The total number of records screened was 494.

**Fig. 2:**
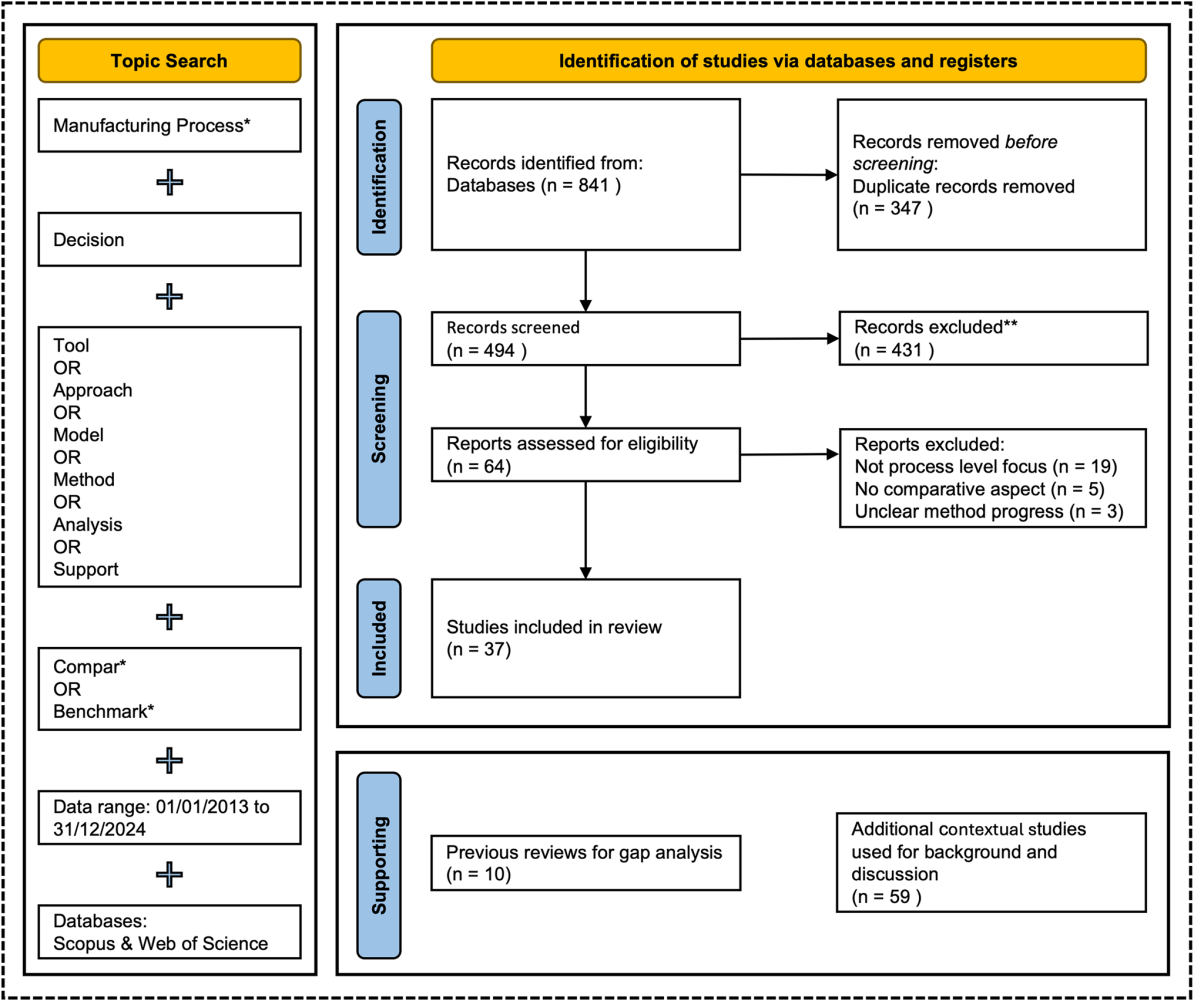
PRISMA Flowchart alongside search terms and scope, indicating the systematically reviewed studies (*n* = 37), previous reviews for gap analysis (*n* = 10), and additional references for context and discussion (*n* = 59)

In addition to the systematically reviewed studies, 10 previous review papers were analysed to assess existing research gaps and validate the novelty of this study’s focus. Furthermore, 72 additional references were used to provide context, theoretical background and discussion support for the identified decision-making tools. While these supplementary references were not part of the systematic selection process, they were essential for comparative analysis and broader sectoral insights. A complete overview of the search terms and the updated PRISMA flowchart is displayed in Fig. 2.

### Study screening and selection criteria

Once the relevant studies were identified, a two-stage screening process was employed to ensure the inclusion of studies most appropriate to the research objective. Studies were considered if they employed cross-sector comparative analysis methods of manufacturing processes. Conversely, studies focusing solely on single-process optimisation without comparison, i.e. production parameter optimisation or those irrelevant to the manufacturing functions, were excluded.

The first phase involved screening titles and abstracts against these criteria. This effectively eliminated a large portion of irrelevant material, with 431 records removed during this stage. The remaining 64 studies were then sought for retrieval for further review. In the second phase, the full text of these studies was thoroughly reviewed using the same inclusion criteria. At this stage, additional emphasis was placed on ensuring that studies demonstrated a clear progression from aim to method to findings, and those lacking sufficient methodological rigour were excluded. This deeper analysis resulted in removing an additional 27 studies, leaving only the most relevant papers that focused explicitly on decision-making tools that incorporate comparative approaches at the manufacturing process level. These two screening phases ensured a refined and focused body of literature directly aligned with the objectives of this research on decision-making tools in the manufacturing sector.

### Data extraction, coding, and analysis

The 37 extracted studies [42–78] were analysed using NVivo to facilitate the categorisation of key concepts and identify patterns and thematic trends across the studies. The process began with a systematic data extraction, where crucial information such as the core concept of the decision-making tool used, the manufacturing process it targeted, and the outcomes reported were identified. This ensured consistency and allowed for a structured examination of how these tools were applied across various industrial settings. To strengthen the objectivity of the qualitative coding, the included studies in NVivo were independently assessed and subjected to a coding review to check for consistency. This process enhanced inter-coder reliability and ensured that the coding framework was applied in a transparent and reproducible manner.

The coding structure was further developed through an iterative review process, which was used to categorise studies by key themes, such as the tools' effectiveness, drivers of functionality and boundaries. This approach facilitated a deeper understanding of the strengths and limitations of each tool, highlighting recurring patterns that revealed the comparative efficacy of various decision-making methodologies. By integrating the data extraction and coding processes, the method comprehensively evaluated the selected studies, ensuring that key insights and trends were analytically captured.

## Findings and discussion

The findings section aims to provide a comprehensive evaluation of the studies included in this review, highlighting key themes, trends, and gaps. The section will present and discuss an analysis of these studies, exploring the evolution of decision-making tools in manufacturing, as well as their impacts, limitations, and intersections among various approaches. Additionally, it will identify gaps in the current research landscape, offering insights for future work.

### Overview of included studies

**Fig. 3:**
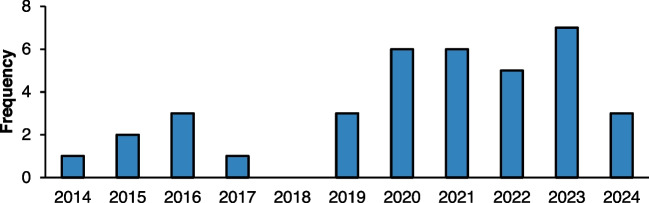
Frequency of analysed studies on decision-making tools for manufacturing processes from 2014 to 2024

An overview of the extracted 37 research papers is provided, highlighting trends in publication frequency over time (Articles summarised in the Appendix). As reflected in Fig. 3, there has been a noticeable increase in the number of studies published over the last decade. Early contributions were relatively scarce, with only one study appearing in 2014. However, since 2019, there has been a consistent rise, with the highest number of publications recorded between 2020 and 2023, demonstrating growing academic interest in the field. This trend suggests a heightened focus on decision-making tools in manufacturing, likely driven by the increasing complexity of industrial processes and the demand for more efficient and sustainable solutions.

Further examination of the literature on decision-making tools revealed various overarching themes, each representing significant dimensions of decision-making in manufacturing. The major themes include “Primary Aim”, “Main Method or Concept”, “Industry or Process Group”, “Main Bases of Comparison”, “Impact Area”, “Operational Level”, “Advantages” and “Limitations”. Each theme encompasses multiple subthemes, offering a comprehensive understanding of the specific focal points of each study. Table 3 provides an overview of the final main themes and their descriptions.

**Table 3:** Overview of key coding categories

Code	Description
Primary aim	Main objectives of decision-making tools
Main method or concept	Core methods or approaches used in decision-making
Industry or Process Group	Industries or processes where tools are applied
Main bases of comparison	Key factors for comparing processes or systems
Impact Area	Areas where tools influence manufacturing outcomes
Operational level	Levels within manufacturing systems targeted by tools
Impacts and Advantages	Positive outcomes and benefits of using decision-making tools
Limitations	Challenges and constraints that limit tool effectiveness

**Fig. 4:**
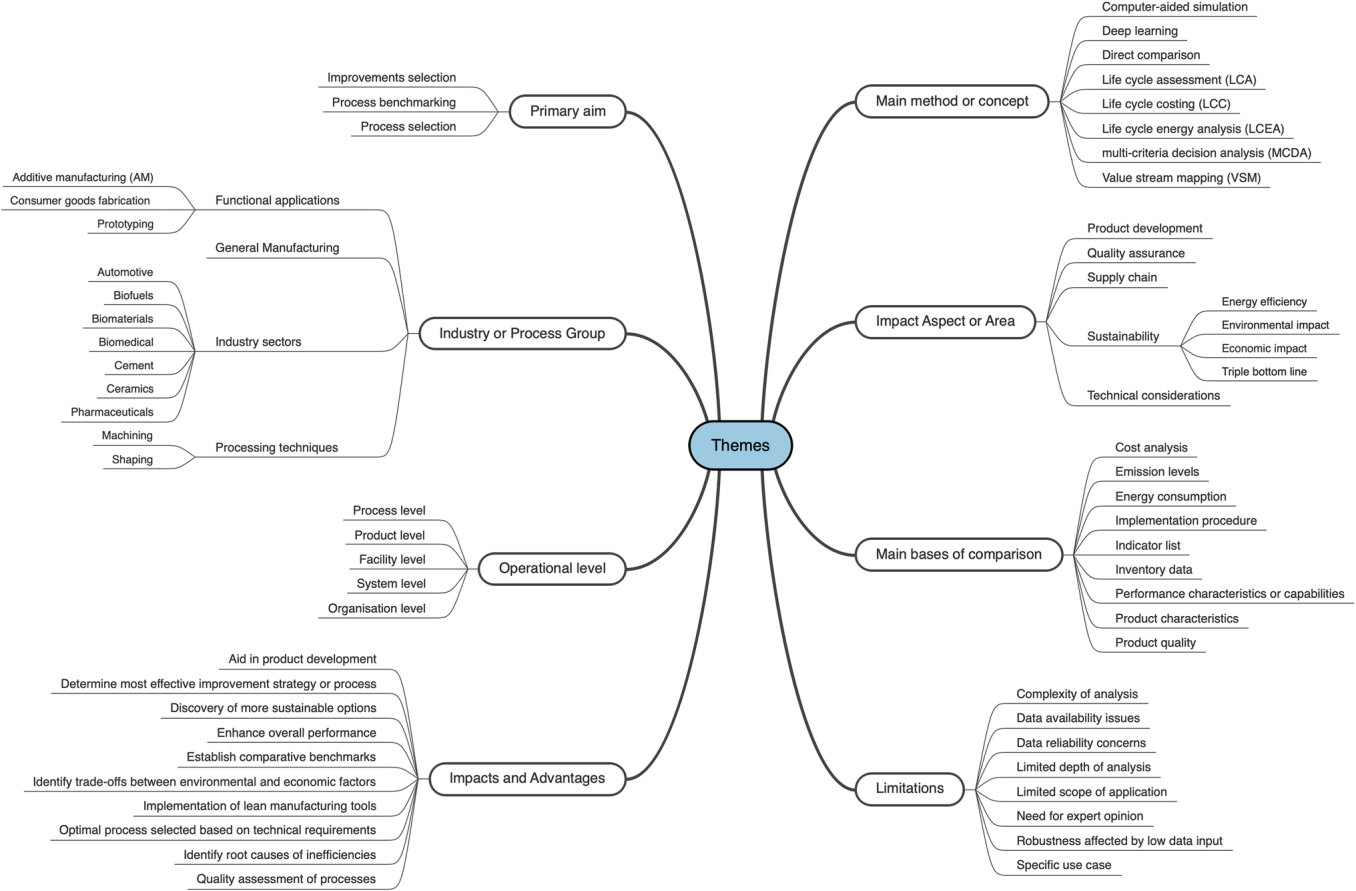
Themes and sub-themes rising from the NVivo coding exercise

The thematic map, shown in Fig. 4, visually represents the key themes and subthemes identified in the review, illustrating their hierarchical structure and interconnections. This aids in clarifying the breadth and depth of the research focus areas, highlighting how decision-making tools have been applied across various manufacturing contexts.

#### Sectoral analysis

The analysed papers covered a broad range of sectors and process groups, with four main categories: generic manufacturing, processing techniques, industrial sectors, and functional applications. Each category reflects different focal points for decision-making tool development and application in manufacturing contexts.

Generic manufacturing refers to research that did not emphasise a specific industry but instead applied general methodologies applicable across multiple sectors. While these studies might not target a single industry, many employed case studies in specific sectors to validate their approaches. This highlights the adaptability and broader applicability of these decision-making tools across various manufacturing contexts. Processing techniques, another significant category, include methods like shaping and machining. These studies often focused on enhancing technical processes and improving production efficiency in areas tied closely to specific processing actions. As these techniques are foundational in many manufacturing industries, tailored data acquisition methods and analysis were necessary for tools to effectively target improvements in these areas. The industrial sectors category includes studies on biomaterials, cement, automotive, and other specialised sectors. These papers tailored their methodologies to meet the technical needs of each industry. Decision-making tools in these studies were developed or adjusted based on sector-specific requirements, such as material characteristics or process constraints, ensuring greater precision in outcomes. The diversity across sectors suggests the importance of customising decision-making tools to align with the unique operational challenges found in each. Lastly, functional applications emerged as the most represented category, particularly in additive manufacturing (AM), which accounts for a significant portion of the research analysed.

**Fig. 5:**
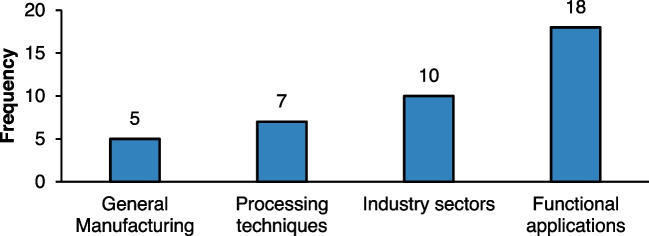
Distribution of industrial focuses of the analysed literature

Other functional applications include prototyping and consumer goods fabrication. This suggests that decision-making tools targeting functional applications are increasingly being shaped by AM technology advancements, focusing on optimising complex processes and improving outcomes in production efficiency, sustainability, and cost-effectiveness [79]. Furthermore, the predominance of AM in this analysis points to its growing importance in modern manufacturing strategies and the necessity of robust decision-making tools tailored to these innovative approaches [80]. Figure 5 illustrates the distribution of studies across these functional and process groups, highlighting the growing focus on functional applications, particularly in AM.

#### Analysis of primary aims

**Fig. 6:**
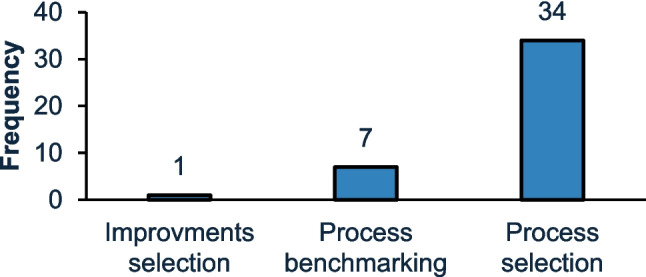
Primary aims represented by the analysed approaches

The analysis revealed three primary aims, namely, process selection, improvement selection and process benchmarking, presented in Fig. 6. The most common focus, by far, was process selection, with a frequency of 34 occurrences. This category involves decisions regarding the choice of manufacturing processes for a product, either in development or already developed. It can also encompass the selection of an optimal manufacturing route, considering the range of available processes. The prominence of process selection aligns with the critical nature of early-stage decisions in manufacturing, where selecting the right processes can significantly affect factors like production efficiency, quality, and cost management [81]. Previous studies have underscored the importance of such decisions, suggesting that early-stage process selection plays a vital role in determining overall operational success [81, 82].

The next most frequent category, process benchmarking, occurred seven times. This category evaluates and compares existing processes against industry standards or best practices to measure performance. It typically involves setting performance metrics and identifying gaps between current operations and ideal benchmarks. This approach is essential for continuous improvement, allowing organisations to gauge the efficiency or sustainability of their processes concerning competitors or established standards. By leveraging benchmarking, companies can identify areas for improvement and implement targeted strategies to enhance performance, thus maintaining competitiveness in rapidly evolving manufacturing landscapes. Process benchmarking offers a data-driven method to assess operational success, often forming the foundation for future process optimisations or innovation initiatives [83, 84].

Lastly, improvement selection was identified only once, suggesting that it is not a commonly studied area within the scope of these papers. This involves choosing strategies to enhance an existing process or system without completely changing the production method. Improvement selection typically reflects incremental changes aimed at process optimisation rather than total overhauls. This finding resonates with the established focus in the industry on continuous improvement methodologies, such as Lean Manufacturing, which prioritise incremental adjustments for ongoing optimisation [85]. Improvement selection, while less frequent than process selection, is crucial for maintaining competitiveness in industries where small efficiency gains can yield substantial long-term benefits [86].

#### Analysis of core methods and approaches

**Fig. 7:**
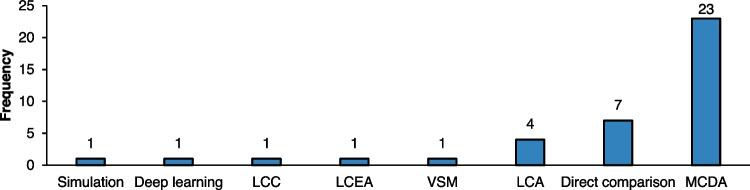
Tools and approaches employed in the analysed literature

The analysis of the core methods and concepts utilised in the reviewed studies revealed a diverse range of decision-making tools with varying frequencies of use, as displayed in Fig. 7. The category with the highest frequency, MCDA, was employed 23 times. MCDA encompasses various techniques to evaluate multiple conflicting criteria in decision-making scenarios. This category included methodologies such as fuzzy logic, Technique for Order of Preference by Similarity to Ideal Solution (TOPSIS) and Analytic Hierarchy Process (AHP). Notably, many studies combined these techniques, integrating two or more methods into a singular decision-making framework tailored to specific needs. This reflects the growing recognition of the complexity involved in decision-making processes within manufacturing, where multiple criteria often need to be evaluated simultaneously [87]. Following MCDA, a direct comparison was utilised in 7 studies, representing a straightforward approach where alternatives are compared against each other based on selected requirements. While effective for specific applications, direct comparison may not adequately capture the nuances of complex decision-making scenarios, especially in environments characterised by interdependent factors and dynamic variables.

Life Cycle Assessment (LCA) was used 4 times to evaluate the environmental impacts associated with all the stages of a product's life, from raw material extraction to disposal. This method aligns with the increasing importance of sustainability considerations in manufacturing decisions as industries strive to minimise their ecological footprint [88, 89]. However, the relatively low frequency suggests that LCA may still be underutilised compared to other methods, which could present an opportunity for further research and development in this area. Other methods, including computational simulations, Life Cycle Costing (LCC), Life Cycle Energy Assessment (LCEA), and Value Stream Mapping (VSM), were each mentioned only once. This infrequent usage indicates that while these methodologies can offer valuable insights into specific aspects of decision-making, they may not yet be fully integrated into the predominant decision-making frameworks utilised in the sector.

Computational simulations and deep learning were utilised once each, signalling their emerging role in process-level decision-making. Simulations offer the advantage of modelling various manufacturing scenarios, enabling decision-makers to predict and optimise outcomes without physical trials. This makes them particularly useful in dynamic manufacturing environments where real-time adjustments are critical. While similarly rare in this analysis, deep learning provides powerful predictive capabilities by learning patterns from large datasets, making it well-suited for more complex and data-intensive decision-making tasks. However, both tools have been applied sparingly, likely due to the technical expertise and computational resources required for their effective implementation [90, 91]. As these technologies continue to advance and become more accessible, their adoption is expected to increase, especially in sectors where precision and adaptability are essential.

#### Analysis of impact areas

**Fig. 8:**
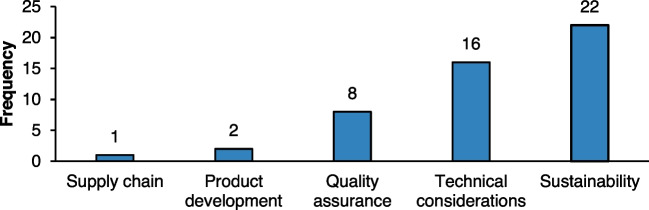
Impact areas covered in the literature

The analysis of the targeted aspects in the reviewed studies indicated a clear focus on several key areas within the decision-making frameworks, displayed in Fig. 8. Sustainability emerged as the most frequently addressed aspect, appearing in 22 instances. This highlights the growing importance of sustainable practices in manufacturing, where decisions increasingly encompass environmental, economic, and social considerations. The sustainability category predominantly focused on environmental and economic impacts, as well as energy consumption and emissions, reflecting the industry's recognition of the need for a balanced approach to resource utilisation and operational efficiency [92]. Technical considerations were the next most frequent, cited 16 times. This aspect involves examining production capabilities, parameters, and the technical specifications of products. It underscores the critical role that technical factors play in decision-making, particularly in ensuring that processes align with the intended operational outcomes and product requirements. This focus on technical specifications suggests that industries are prioritising both efficiency and the feasibility and excellence of the manufacturing processes. Quality assurance was addressed in 8 studies, indicating its significance in the decision-making process. Quality assurance pertains to the systematic processes to ensure that products meet defined quality standards. The presence of this aspect in numerous studies reflects the industry's commitment to maintaining high-quality outputs, which is essential for competitiveness and customer satisfaction. Product development, mentioned twice, focused on selecting processes and materials based on the specific product under development. This aspect emphasises the importance of aligning production methods with product characteristics, capabilities, and material availability. The limited frequency of this category compared to others may suggest that while product development is crucial, it may often be integrated into broader considerations of sustainability and technical factors within decision-making frameworks [93]. Lastly, the minimal focus on the supply chain, identified only once, indicates a potential area for further exploration in future studies. Given that supply chain dynamics play a critical role in manufacturing operations, more comprehensive analyses could yield valuable insights into how decision-making tools can be applied effectively across the entire supply chain [94].

Overall, the results reflect an industry-wide trend toward integrating sustainability and technical considerations into decision-making processes, with a clear recognition of their impact on overall performance and competitiveness which aligns with conclusions from previous studies [95, 96].

### Thematic evolution

**Fig. 9:**
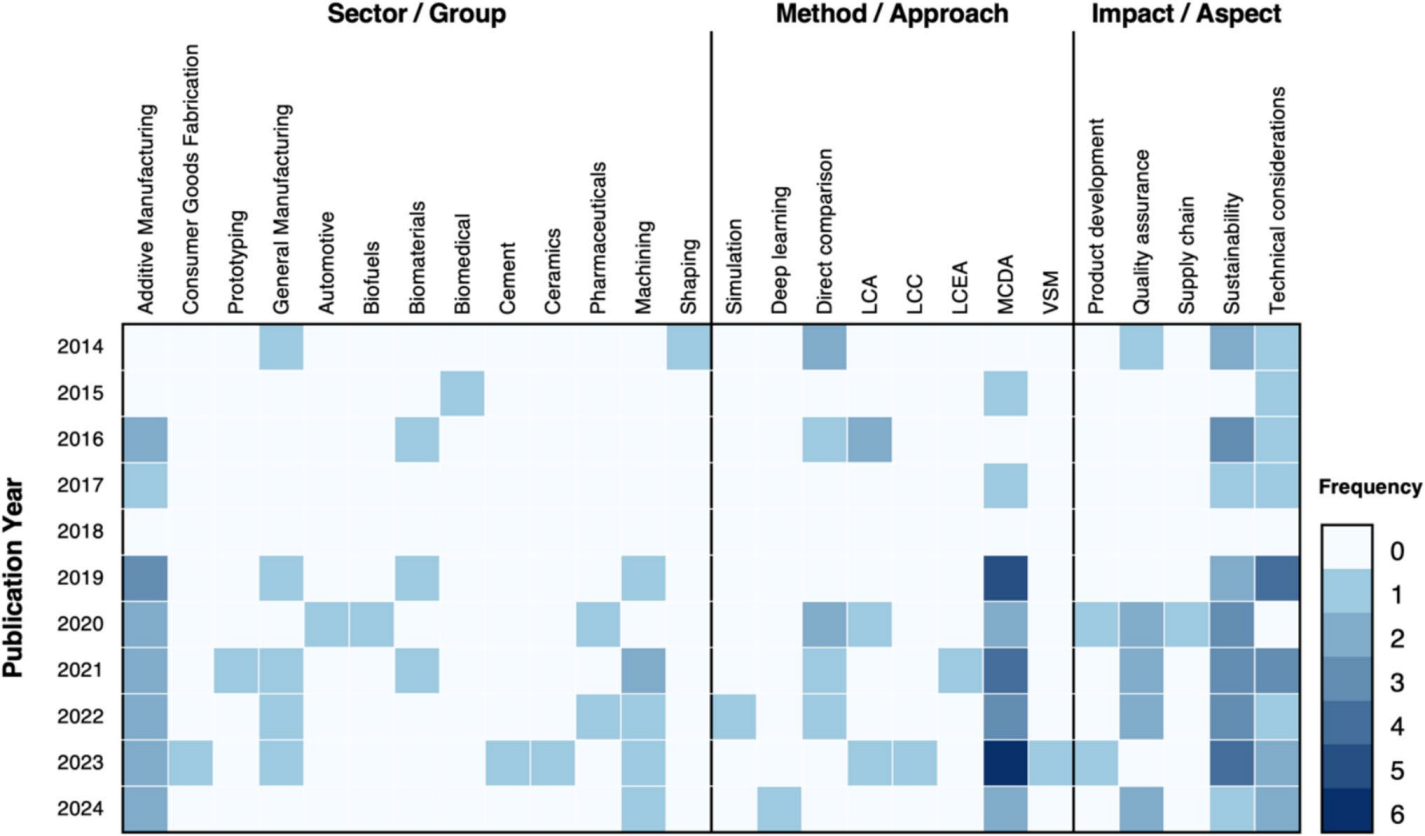
Thematic evolution heatmap

The evolution of decision-making tools in manufacturing can be traced by examining the chronological progression of research across sectors, methods, and impact areas. This subsection explores the development trends of these tools, revealing key milestones and shifts in focus within the literature. The use of diverse methodologies has expanded in parallel with emerging industry needs, technological advancements, and an increasing emphasis on sustainability and efficiency. The heatmap in Fig. 9 provides a detailed view of the evolution by mapping the publication year against the industry sectors, methods employed, and impact areas considered in each study. This visual representation reveals several important trends over time.

Regarding the sectoral focus of the studies, there has been a consistent increase in academic interest in AM, largely due to its superior flexibility and customisation potential, which allows for complex geometries and reduced material waste [97]. Another area of growing interest is machining processes, while papers focusing on more generic manufacturing methods remain the third most prevalent. It is worth noting that, contrary to prevailing expectations, sectors such as biomaterials and biofuels have not been as prominently featured. This could be attributed to the need for further technological development and refinement of these products before decision-making tools can be effectively applied to their utilisation [98]. Other sectors and groups are more scattered across the timeline, suggesting a more sporadic academic interest or niche focus in certain areas.

Regarding the methods and approaches utilised, MCDA has seen a notable increase in application, particularly in 2023. This is likely due to its ability to handle the various and often conflicting factors involved in manufacturing decision-making, such as cost, efficiency, and sustainability. Direct comparison has also been consistently used over the years, possibly because of its simplicity and the straightforward ability to contrast available options. While recognised for their analytical power, tools like deep learning and computational simulation have only started gaining traction more recently, particularly for process-level decisions. This shift likely reflects advancements in computational technology, making these tools more accessible and applicable in real-time decision-making.

In terms of impact areas, sustainability and technical considerations have maintained a strong presence throughout the period, likely due to the increasing global emphasis on environmental concerns and the push for more efficient, technologically advanced manufacturing processes. Quality assurance, however, has only gained significant traction in the past four years, highlighting the growing recognition of the need for consistent product quality as markets become more competitive and customer expectations rise. This trend underscores a shift in focus from just optimising production to ensuring that the end products meet increasingly stringent standards.

### Cross-thematic analysis

The intersections of the various key parameters of the examined studies have been explored. This analysis provides key insights into how specific decision-making approaches contribute to evaluating manufacturing processes and how those evaluations ultimately influence the criteria for comparison.

#### Interactions between impact areas

**Fig. 10:**
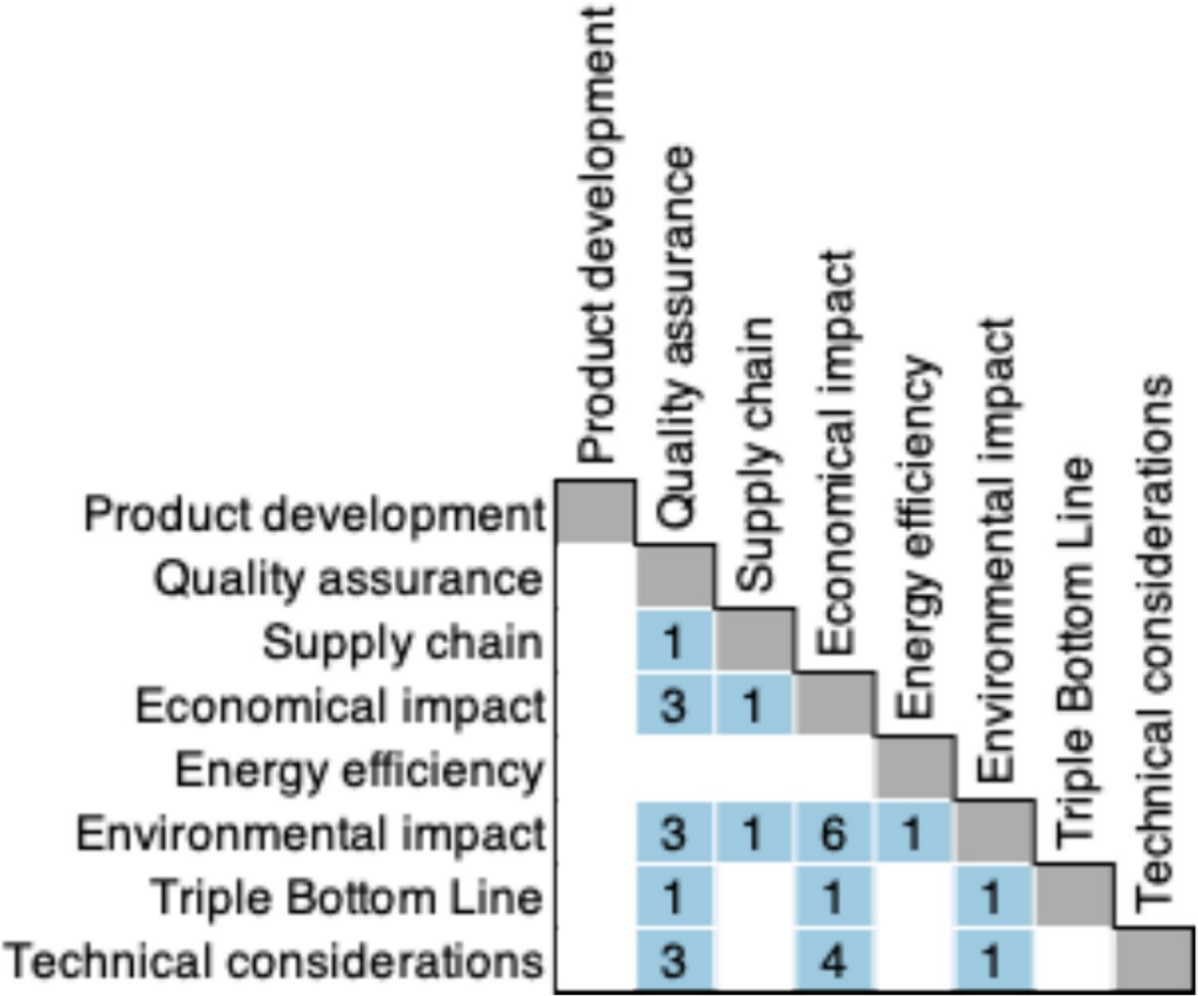
The interactions of impact areas with one another

The matrix presented in Fig. 10 provides a detailed view of the interactions between various impact areas in decision-making tools for manufacturing. It reveals how different aspects are connected and how they have been considered in the analysed studies.

One notable finding is the prominence of quality assurance and environmental impact as key focal points across multiple intersections. Quality assurance shows significant interactions with other impact areas, including economic impact and technical considerations. This suggests that ensuring product quality is not an isolated concern but is deeply interwoven with cost efficiency and the technical feasibility of processes. Similarly, the frequent intersections between environmental impact and areas like economic impact and quality assurance emphasise the growing importance of sustainability, with many studies focusing on minimising environmental footprints while maintaining product standards and managing costs.

The central role of economic impact in the matrix further highlights the critical balance between financial considerations and other decision-making factors. The economic impact frequently appears across the matrix, particularly with environmental impact and technical considerations. This indicates that cost analysis is pivotal when evaluating environmental and technical outcomes, showcasing the increasing need for integrated approaches that combine financial viability with sustainability goals.

In contrast, supply chain considerations show relatively limited intersections with other impact areas. While they are linked to quality assurance, economic impact, and environmental impact, these connections are less frequent. This could indicate that supply chain management, though important, is often treated separately from immediate process-level decisions. However, with the growing complexity of global supply networks, future studies may need to focus more on integrating supply chain impacts into the broader decision-making framework.

Technical considerations demonstrate strong connections with multiple areas, particularly economic impact and quality assurance, further underscoring the importance of technical feasibility in the decision-making process. The significant frequency of interactions involving technical considerations suggests that many studies prioritise technological factors as essential criteria, especially when evaluating manufacturing processes’ potential economic and quality outcomes.

Moreover, the Triple Bottom Line, a context for balancing environmental, social, and economic factors [99], is primarily connected to environmental and economic impact. This reinforces the idea that sustainability, while multidimensional, is often approached through the lens of environmental and economic outcomes, particularly in manufacturing sectors that aim to reduce emissions, energy consumption, and waste. The social aspect is not considered solely but only as part of a holistic insight.

This cross-analysis of impact areas reveals a complex interplay between economic, environmental, and technical considerations in decision-making processes. While traditional factors like cost and quality remain central, there is a clear trend towards integrating sustainability, mainly through the lens of environmental impact and energy efficiency. However, the relatively lower focus on supply chain interactions suggests that further integration of logistics and distribution processes into decision-making tools could enhance their comprehensiveness in future studies.

#### Method, impact area and the bases of comparison

**Fig. 11:**
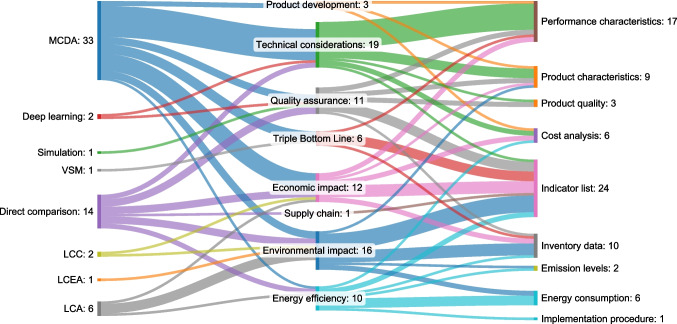
Flow from decision-making methods to impact areas and then to bases of comparison, presented using a Sankey diagram (the numbers represent the quantity of connections)

The tools and approaches were analysed in terms of the impact areas they address, and these impact areas were further examined against the bases of comparison applied in the assessments. The combined relationships are displayed in Fig. 11 as a Sankey diagram, providing a comprehensive overview of the flows from decision-making methods to impact areas and then to bases of comparison.

##### Correlation of methods and impact areas

The analysis of methods and their corresponding impact areas reveals key patterns in the application of decision-making tools across different objectives. Among the reviewed studies, MCDA stands out as the most versatile method, applied across a wide range of impact areas. It is particularly prominent in technical considerations. This indicates its adaptability in scenarios where trade-offs between multiple, often conflicting criteria are necessary. MCDA's application in sustainability-related areas further underscores its strength in integrating economic, environmental and technical factors, which is critical when integrating sustainability aspects in modern manufacturing contexts. Previous studies have indicated that MCDA is well-suited for environments characterised by uncertainty and the need for stakeholder involvement, reinforcing its relevance in contemporary manufacturing contexts [100, 101].

LCA is primarily associated with environmental and energy efficiency considerations, reflecting its traditional role in assessing the environmental footprint of processes and materials. Its strong focus on these impact areas aligns with the increasing demand for sustainability assessments in production systems. Similarly, LCC is used for evaluating economic impacts, indicating its utility in cost-based sustainability assessments, where economic efficiency is weighed against environmental benefits over the life cycle of products and processes. While LCA has often been applied independently, LCC was used in conjunction with LCA, allowing the two methods to complement and address each other’s limitations[102]. LCEA, a more specialised life cycle technique focusing specifically on energy, has also emerged as an option for evaluating the energy consumption and efficiency of manufacturing processes. This approach allows for a deeper analysis of energy-related impacts, complementing broader sustainability assessments conducted through LCA.

Direct comparisons also show a focused but distinct pattern in their application. For instance, it appears relatively balanced across economic, environmental, technical and quality assurance impacts. This indicates its utility in straightforward benchmarking tasks where trade-offs between different criteria must be quickly compared. Interestingly, more advanced methodologies, such as deep learning and computational simulations, are beginning to emerge, linking directly to quality assurance and technical considerations. This suggests a shift towards adopting innovative computational techniques that enhance precision and optimise manufacturing processes. Incorporating these advanced methods indicates an industry trend toward leveraging technology to improve decision-making capabilities and operational outcomes.

One notable pattern is the relatively low occurrence of VSM, with its singular connection to the Triple Bottom Line, addressing environmental, economic, and social aspects of sustainability. In the instance where VSM was applied, it was used to identify root causes of inefficiencies in a manufacturing organisation and paired with MCDA to guide improvement strategies. This suggests that while VSM is highly effective in diagnosing inefficiencies within broader systems, it is less commonly employed for individual process evaluations than more adaptable methods like MCDA or LCA, which are more frequently used across specific impact areas and goals.

##### Correlation of impact areas and bases of comparison

From the identified impact areas, several bases of comparison emerge. Technical considerations, environmental impact, and quality assurance are frequently evaluated based on performance characteristics, product quality, and various indicators that reflect specific and measurable criteria for assessing the effectiveness of manufacturing processes. Technical aspects are mainly linked to performance characteristics, underscoring the industry's ongoing priority of optimising operational efficiency and process accuracy. On the other hand, environmental impacts are assessed more broadly through energy consumption, emission levels and inventory data, further emphasising the growing importance of sustainability in decision-making.

Economic impacts are typically assessed through cost analysis and indicator lists, reflecting the financial considerations integral to manufacturing decisions. Similarly, energy efficiency, though less frequently addressed, ties directly to energy consumption and emission levels, aligning with the sector’s increasing emphasis on reducing energy use and emissions. Though less prominent, supply chain considerations focus on the use of indicator lists, suggesting that supply chain metrics are still an emerging focus in integrating decision-making tools.

Additionally, the diversity in bases of comparison indicates that decision-making tools are not only about optimising one aspect but also managing complex trade-offs between various objectives, such as cost versus sustainability or quality versus efficiency. The link between emerging technologies like deep learning and traditional aspects like quality assurance suggests a gradual shift towards smarter, data-driven approaches to manufacturing decisions. The industry’s growing reliance on integrated, multi-faceted methodologies like MCDA and the application of advanced technologies point towards the future of decision-making tools, where processes are evaluated holistically, with equal consideration for economic, environmental, and technical factors. This approach enables businesses to make more informed and balanced decisions, leading to more sustainable and efficient manufacturing practices. For companies looking to adopt these tools, it is essential to understand the immediate process and how different methods, impact areas, and comparison metrics interact to inform the best course of action.

Consequently, a prominent pattern is the central role of the indicator list, which shows significant interactions across multiple areas. With the highest frequency of occurrences, utilising an indicator list with a wide span of evaluation points underscores the importance of a comprehensive outlook enabling the evaluation of various performance aspects. This suggests that a well-defined set of indicators can be foundational in assessing manufacturing processes, ensuring that critical factors are systematically and holistically evaluated [103–105].

The interactions involving performance characteristics or capabilities, as well as product characteristics, also stand out. The high frequency of connections to performance metrics indicates that manufacturers prioritise assessing their production capabilities to ensure alignment with market demands and technical specifications. This focus on performance highlights the critical need for manufacturers to continuously evaluate their operational capacities and adapt to evolving industry standards [106]. Interestingly, the data reveals a lesser emphasis on implementation procedures. This indicates a potential gap in practical applications, where the theoretical understanding of processes does not always translate into effective execution.

The integration of methods, impact areas and bases of comparison highlights the nuanced and multi-dimensional nature of decision-making in manufacturing. MCDA emerges as the most versatile method, capable of addressing various technical, economic and environmental factors. The emphasis on these impacts underscores the industry’s dual focus on optimising operational performance while adhering to sustainability goals.

#### Methods across operational levels

**Fig. 12:**
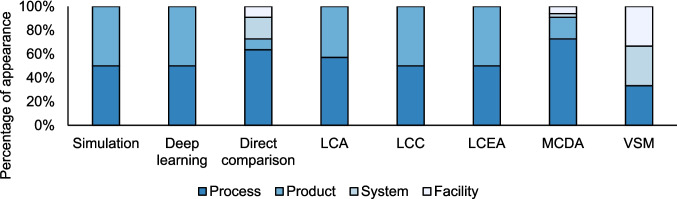
Distribution of appearances of different decision-making approaches across operational levels in manufacturing systems, as reported in the literature. The percentages represent the distribution of instances where each approach is applied at the process, product, system, and facility levels. These appearances may occur across multiple levels within the same study or in different studies

The analysis of decision-making techniques across different operational levels (facility, process, product and system) reveals distinct patterns in their usage, as presented in Fig. 12.

Specific approaches, like Direct Comparison, demonstrate flexibility by being applied at the process level and scaling up to facility or system levels when needed. For example, performance analysis at the process level can inform broader decisions, such as facility-wide operational strategies or overall system efficiency. Additionally, some methods can simultaneously address multiple operational levels within the same study. For instance, a method applied to the process level may also aggregate data to enable comparisons or decisions at higher levels, such as system-wide evaluations [107].

Similarly, MCDA is adaptable across different operational levels, making it useful for detailed process and higher-level system assessments. However, this scaling can be complex, requiring additional layers of data, broader stakeholder engagement, and more comprehensive system-wide modelling, and therefore, it is more limited. However, methods such as LCA, LCC, and LCEA, which focus on environmental and cost assessments, are generally limited to the process and product levels. These tools are practical within their specific scope but are more challenging to apply at the facility or system levels due to their specialised focus on isolated processes or products. VSM demonstrates that it was applicable for operational improvements at the facility and system levels, indicating that its focus on optimisation at the process level can be translated upwards. This is due to the intrinsic nature of the approach [108].

Conversely, tools like computational simulations and deep learning are evolving methods used primarily for product and process analysis. Expanding their application to larger, more complex systems or facilities presents challenges due to the computational power and extensive modelling required for broader-scale use. While these tools offer significant potential for process optimisation, scaling them for facility-wide or system-level analyses while maintaining granularity is often complicated and resource-intensive.

In summary, while some tools can scale from process to facility or system levels, the complexity of this transition varies. Tools like MCDA and Direct Comparison offer more flexibility, while others like LCA and LCC remain constrained to process-level analysis. Applying tools across multiple operational levels is critical for achieving comprehensive decision-making in complex manufacturing environments, but this remains a challenge for many methodologies.

#### Analysis of advantages and limitations of identified methods

**Fig. 13:**
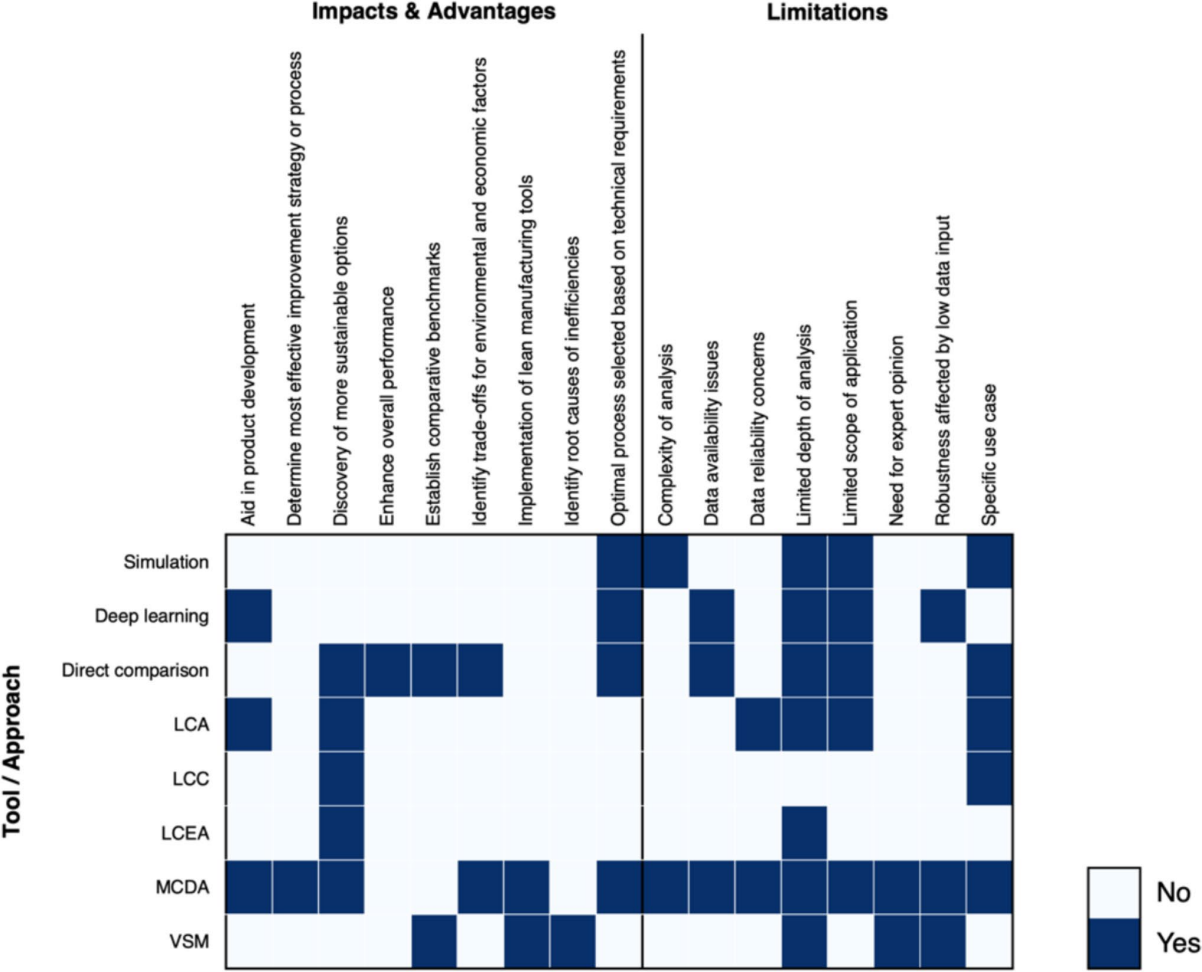
Advantages and limitations of the identified approaches

The analysis of the advantages and limitations of the various methods and approaches used in decision-making for manufacturing, as visualised in the matrix in Fig. 13, highlights some significant trends.

##### Advantages

MCDA emerges as the most widely applicable tool, exhibiting strengths across several areas such as product development, process improvement and identifying sustainable options. This aligns with existing literature highlighting MCDA’s ability to synthesise multiple criteria in complex decision-making environments, making it particularly valuable in manufacturing contexts where trade-offs between technical and sustainability criteria are crucial [109]. Similarly, LCA was shown to aid significantly in identifying environmental impacts and establishing environmental benchmarks. This aligns with its well-established role in sustainability assessments, particularly its capacity to quantify environmental burdens across a product's lifecycle [110]. Although more limited, the presence of LCC and LCEA reflects their increasing relevance in economic and energy efficiency assessments, respectively. LCC is particularly important for cost-based sustainability assessments, where long-term financial considerations are weighed against environmental outcomes, often in conjunction with LCA to overcome mutual limitations. LCEA, though less frequently utilised, offers a more pinpointed focus on energy consumption and efficiency, which is becoming critical in the age of energy-conscious manufacturing [111].

Direct Comparison appears as an advantageous method when benchmarking between alternative strategies or processes is required. Its primary utility lies in enhancing overall performance by establishing clear comparisons between alternatives. It is critical for manufacturers seeking to optimise processes or choose between different materials, technologies, or production strategies. While direct Comparison lacks the depth to comprehensively address trade-offs across multiple sustainability dimensions, it provides significant value when a single metric or aspect is a central focus. Its effectiveness in comparative evaluation allows manufacturers to make more informed decisions based on competitive benchmarks and performance data without requiring complex modelling or extensive data inputs. This method could be further improved by incorporating weighted criteria, allowing for a more nuanced evaluation that considers the relative importance of different performance factors. By doing so, direct comparison could extend its utility beyond single-metric assessments, enabling manufacturers to balance competing objectives such as cost and environmental impact. This would allow for a more flexible and robust decision-making process, particularly in industries with crucial trade-offs between these factors.

A notable advantage of VSM is its unique capability for root cause analysis and identifying areas for improvement in manufacturing systems, demonstrated in its combined use with MCDA. While limited in standalone applications, its utility in diagnosing inefficiencies is well-recognised, particularly in lean manufacturing environments [112]. Although not as widely used as other tools, Deep Learning and Simulations offer strong potential for performance improvements by enhancing the predictive accuracy of models, which is crucial for quality assurance and technical considerations. These emerging tools, especially Deep Learning, reflect ongoing shifts toward data-driven decision-making, gaining traction as manufacturing processes become more digitised under Industry 4.0 and the recently coined, industry 5.0 [113, 114].

##### Limitations

Despite these advantages, the heatmap also reveals critical limitations for each tool, some of which align with documented challenges in the literature. MCDA, while offering robust decision support, is constrained by several key limitations. These include the complexity of analysis, data reliability issues, and weighting inconsistencies. Additionally, the absence of a discussion or review process to interpret the results can lead to oversimplified recommendations. The assumed preferential independence between criteria can further distort results, especially when interactions between criteria are not considered. Moreover, incorporating costs as a criterion often fails to capture opportunity costs adequately, leading to suboptimal resource allocation [115, 116]. These challenges highlight the need for more nuanced approaches to applying MCDA effectively in manufacturing decisions.

LCA and LCC face similar challenges. Both tools, while comprehensive, suffer from high data demands and complexity in analysis, mainly when applied to diverse manufacturing processes. Studies have pointed out that the reliability of LCA results can be heavily influenced by the scope of the analysis and the assumptions made during data collection, which can limit its broader applicability in real-world manufacturing contexts [117]. LCEA, while less complex than LCA, shares data availability concerns, especially as it focuses exclusively on energy metrics without considering broader environmental or social factors.

In contrast, VSM and Deep Learning exhibit more narrow application scopes. VSM's use primarily concerns identifying waste and inefficiencies within existing systems rather than offering comprehensive decision support across broader impact areas. This aligns with its traditional role in lean manufacturing, which focuses on process streamlining rather than evaluating sustainability or economic trade-offs. Deep Learning, while showing promise, is constrained by the robustness of its results, mainly when low-quality or limited data is available. Moreover, once a model has been established for a specific task, it will require many data points and further training to enable its adaptation to other projects, limiting its expansion. These challenges are well-documented in the literature on AI-driven decision tools [118–120].

Direct Comparison, while effective in benchmarking and enhancing overall performance, demonstrates limitations due to its finite use cases. It is often restricted to comparative evaluations and lacks the flexibility to offer deeper insights into long-term strategic improvements or sustainability metrics. This limitation means direct comparison is typically used for isolated decision points or for improving specific, measurable areas rather than facilitating broader assessments of multi-dimensional challenges.

#### Discussion and implications

The findings suggest a clear pattern: methods with broader applicability, such as MCDA and LCA, offer more comprehensive decision-making support, particularly in balancing sustainability and technical performance. However, they also face significant limitations related to data complexity and the depth of analysis required. This supports the argument that while these tools provide valuable insights, their practical implementation in manufacturing contexts requires careful consideration of data availability, skill requirements and the specific goals of the analysis.

Conversely, while highly effective in specific contexts, tools with narrower scopes, such as VSM, Direct Comparison, and Deep Learning, are not yet as widely applicable across multiple dimensions of manufacturing decision-making. This reflects a broader trend in the field where newer, more specialised tools, though promising, still require further refinement to broaden their use cases. Future research should explore integrating these emerging tools into more comprehensive decision-making frameworks to capitalise on their strengths without sacrificing the breadth of analysis.

Additionally, the combined use of tools like VSM with MCDA suggests a trend toward hybrid approaches that could mitigate some of the limitations observed with individual tools. This opens up a potential area for future studies to investigate the efficacy of tool combinations in addressing multi-criteria decision-making challenges, especially in sustainability assessments and process improvements.

### Synthesis of findings

This section synthesises findings across decision-making tools and their impacts at the process level. The tools differ in their scope, complexity, ease of use, and applicability across industries, with each addressing distinct aspects of process-level decision-making. To support structured comparison, Table 4 defines the attributes used for evaluation, and Table 5 reports relative ratings of each tool against these attributes.

**Table 4:** Attributes used to analyse the tools and approaches

Attribute	Description
Operational scope	How easily the tool can be scaled or applied across different operational levels (from process to facility level), defined by the reported use cases
Technical complexity	The level of technical or analytical difficulty required to implement and use the tool, defined by modelling depth, algorithms or any specialist expertise required
Integration potential	The ability of the tool to be integrated into existing systems or processes, particularly in a digital or automated manner, defined by any previous documented applications
User-friendliness	The simplicity and usability of the tool for non-specialists or practitioners with minimal training or setup required
Data requirements	The amount and complexity of data needed to use the tool effectively
Trade-off analysis	The tool’s ability to consider and balance multiple, potentially conflicting criteria

**Table 5:** Summary rating of each tool or approach based on various attributes

Approach	Operational Scope	Technical Complexity	Integration Potential	User-Friendliness	Data Requirement	Trade-off Analysis
Simulation	★★★☆☆	★★★★☆	★★★☆☆	★☆☆☆☆	★★★★★	★★★☆☆
Deep learning	★★☆☆☆	★★★★★	★★★★☆	★☆☆☆☆	★★★★★	★★★☆☆
Direct comparison	★★★★☆	★★☆☆☆	★★☆☆☆	★★★★★	★★☆☆☆	★★☆☆☆
LCA	★★☆☆☆	★★★★☆	★★★☆☆	★★★☆☆	★★★★★	★★★★☆
LCC	★★☆☆☆	★★★☆☆	★★★★☆	★★★☆☆	★★★★☆	★★★★☆
LCEA	★★☆☆☆	★★★☆☆	★★☆☆☆	★★★☆☆	★★★☆☆	★★★☆☆
MCDA	★★★☆☆	★★★★★	★★★☆☆	★★☆☆☆	★★★☆☆	★★★★★
VSM	★★★☆☆	★★★☆☆	★★☆☆☆	★★★★☆	★★☆☆☆	★☆☆☆☆

★ indicates a higher rating and ☆ indicates a lower rating

The ratings were assigned using consistent qualitative criteria drawn from reported applications. Broader applicability across operational levels (operational scope), greater modelling or specialist demands (technical complexity), demonstrated integration into digital or automated systems (integration potential), lower training burden (user-friendliness), larger or higher-quality data needs (data requirements), and explicit balancing of multiple, potentially conflicting criteria (trade-off analysis).

These ratings are intended as heuristic indicators rather than precise quantitative scores. The approach reduces subjectivity by applying the same criteria across all tools, while acknowledging that the results remain indicative and should be interpreted in context.

Simulation and deep learning are powerful tools with high data requirements and technical complexity, excelling in detailed process modelling and advanced analytics. However, they are less user-friendly, requiring specialised expertise and substantial infrastructure, limiting their wider application across industries. In contrast, Direct Comparison is a more straightforward, more accessible tool with low complexity and minimal data needs, making it ideal for quick benchmarking. Its high user-friendliness allows rapid deployment, but it is less effective for complex, multi-tiered analysis across different variables. LCA and LCC offer in-depth sustainability and cost analyses, though both require significant data and are limited in operational scope. While these tools provide robust trade-off analyses, their complexity restricts broader usability without sufficient expertise and resources. MCDA provides a structured approach to decision-making that is well-suited for complex, multi-criteria scenarios, but it requires a time-intensive setup and is not easily executable. Meanwhile, VSM is cost-effective and user-friendly, best suited for identifying value-adding steps and eliminating waste. However, its effectiveness might be limited across impact areas without customisation or integration with additional tools.

The synthesis of findings underscores the importance of selecting decision-making tools based on the specific needs of the manufacturing process and the desired impact areas. Tools like Simulation, deep learning, and MCDA are ideal for comprehensive, data-heavy evaluations but require significant expertise and infrastructure. More straightforward tools like Direct Comparison and VSM offer easy-to-use functionality for targeted applications but lack the versatility of more complex tools. As manufacturing processes evolve, particularly under the influence of Industry 4.0 and sustainability imperatives, a hybrid approach that leverages multiple tools in tandem may offer the best pathway to balancing trade-offs and achieving long-term sustainability goals.

## A practical guide for effective integration of decision-making tools in manufacturing settings

Despite the significant strides made in developing decision-making tools for manufacturing processes, crucial gaps still limit their full utilisation. Among these is the lack of adoptability to emerging Industry 4.0 technologies like IoT, AI, and real-time data processing. Furthermore, insufficient attention has been given to integrating these tools into fully functioning manufacturing environments. Many studies overlook key steps required to smoothly incorporate decision-making tools within operational systems, from data acquisition and sharing to real-time processing and long-term monitoring. Furthermore, strategic alignment, system-wide incorporation and skill requirements are rarely discussed. This gap is evident in the fact that none of the covered literature provided clear guidance on how these tools can be seamlessly integrated into a real-world manufacturing context.

**Fig. 14:**
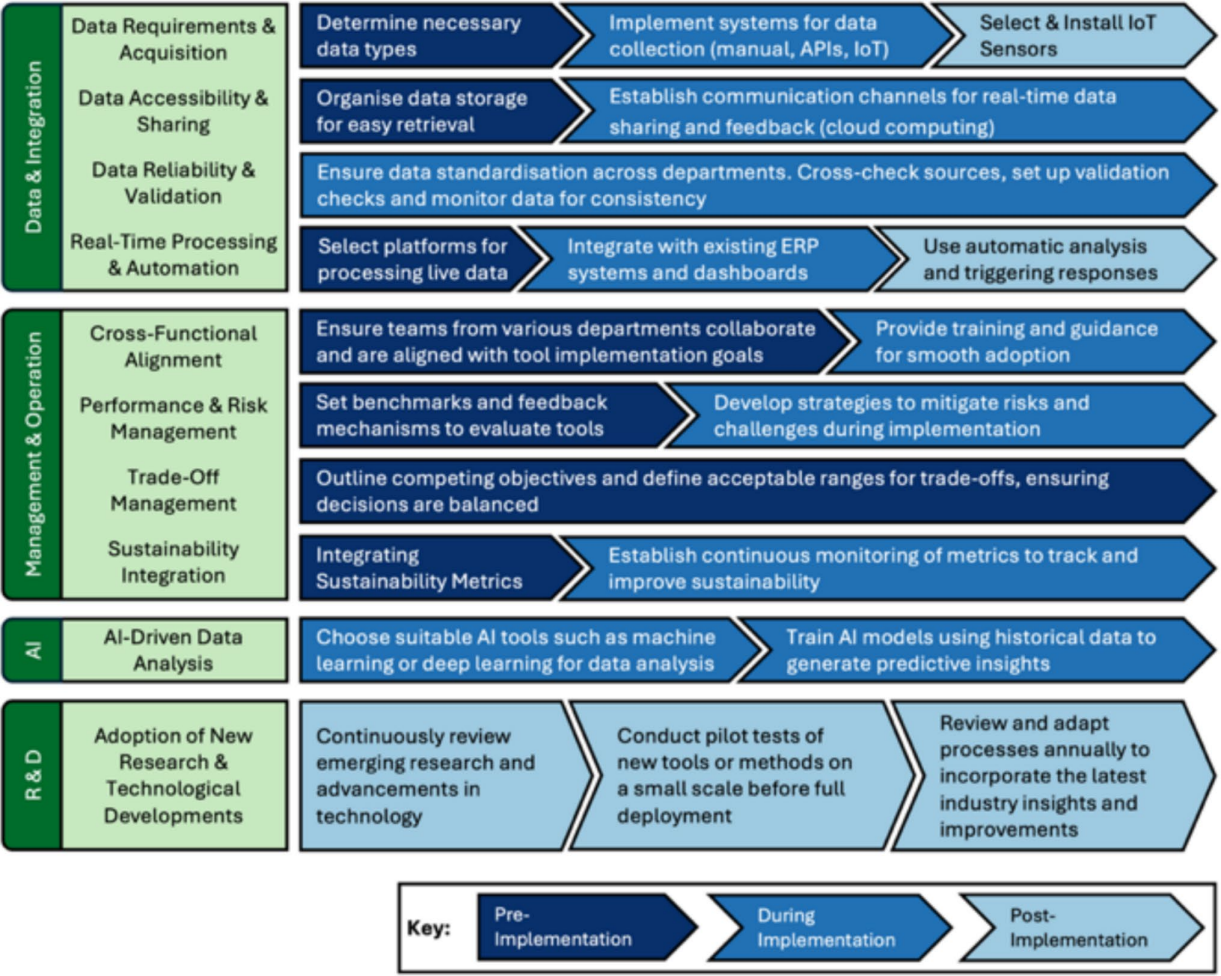
Structured guidelines for decision-making tool integration in manufacturing systems, highlighting phased implementation

In response to these gaps, a structured guide has been developed to facilitate the smooth integration of decision-making tools into manufacturing systems, presented in Fig. 14. This step-by-step process emphasises four critical areas: Data & Integration, Management & Operations, AI, and Research & Development. Each area is subdivided into key actionable steps spanning three phases: pre-implementation, during implementation, and post-implementation. In which each phase has a specific purpose, pre-implementation establishes the conditions and requirements for adoption. Implementation delivers the systems, processes, and training needed for operation. Post-implementation ensures monitoring, refinement, and scalability. By aligning these phases with specific tasks in data handling, technology integration, workforce preparation, and leadership oversight, businesses can ensure a structured transition from planning to execution. The guide, therefore, functions both as a visual roadmap and as a practical framework that links the gaps identified in the review to actionable steps supported by insights from recent research.

### Data management and tool integration

This area of the guide addresses how data handling should progress through the three phases: establishing inventories and structures in pre-implementation, creating live pipelines during implementation, and ensuring automated monitoring and refinement in post-implementation.

Effective data management is essential for the successful use of decision-making tools. In the pre-implementation phase, the priority is identifying the necessary data types and ensuring data storage systems are organised for easy retrieval. Establishing data accessibility and sharing mechanisms is equally important at this stage, so that different departments can contribute to and draw from a common data infrastructure. These recommendations directly respond to the review finding that many methods, especially LCA and LCC, face high data demands and reliability concerns (Fig. 13), which limit their broader adoption.

During implementation, businesses should focus on establishing data acquisition and real-time data-sharing mechanisms, such as cloud platforms, to facilitate continuous communication between departments [121]. Data validation and standardisation must also be prioritised to ensure consistency across the organisation [122]. Moreover, at this stage, integration with existing enterprise platforms, including ERP systems and dashboards, supports visibility across operations. This aligns with evidence from the review that tool scalability across operational levels (Fig. 12) is contingent on effective data pipelines. In the post-implementation phase, installing IoT sensors tailored to ongoing data collection needs will allow continuous monitoring and refinement of decision-making processes [123]. Automation capabilities can be introduced to trigger responses directly from live data, further embedding decision-making tools within the production environment. Maintaining robust validation processes and data governance ensures that the system continues to generate reliable insights. The progression from basic data organisation to automated real-time integration ensures that decision-making tools are not only implemented successfully but also sustained and adapted as operational needs evolve.

### Managerial and operational preparation

This area outlines how organisational readiness develops across phases, beginning with alignment of roles and objectives, continuing with training and procedures during implementation, and closing with review and continuous improvement in the post-implementation stage.

Cross-functional alignment is critical to the successful adoption of decision-making tools. In the pre-implementation phase, all departments must be aligned with the implementation goals to avoid fragmentation or miscommunication [124]. Ensuring that operations, IT, and management team members work cohesively is essential. Moreover, benchmarks should be set to evaluate the tools' impact and prepare feedback mechanisms for ongoing assessments. This reflects the review’s observation that while tools such as MCDA offer robust trade-off analysis, they are constrained by weighting inconsistencies and the absence of systematic review processes (Fig. 13), underscoring the need for organisational procedures to interpret and act on outputs.

As implementation progresses, comprehensive training should be provided to ensure all employees are proficient with the new tools. Training should focus not only on using the tools but also on understanding their implications for decision-making. Risk mitigation strategies should also be developed to address potential challenges, ensuring the smooth integration of tools without disrupting ongoing processes [125]. Managing trade-offs is another key area of focus. Businesses should identify competing objectives and define acceptable ranges for trade-offs [126]. For example, companies may need to decide how much cost can be sacrificed for environmental gains. By embedding alignment, training, risk management, and trade-off handling within a phased approach, decision-making tools can be integrated in a way that strengthens organisational cohesion and delivers sustained impact. These practices are directly tied to the review’s synthesis that hybrid approaches and multi-criteria frameworks (Table 5) require managerial structures to balance complexity with usability.

### AI integration

This part of the framework sets out the AI use cases and how models are developed and embedded during implementation.

AI is central to modern decision-making, offering advanced capabilities for analysing data, predicting outcomes and optimising manufacturing processes [127]. Businesses should identify specific decision-making challenges AI can address, such as predictive maintenance, energy optimisation or production scheduling [128–130]. Selecting suitable AI models (e.g., machine learning, deep learning) based on data availability and complexity is critical. Preparing high-quality, standardised datasets is essential for effective model training. Subsequently, AI models should be trained using historical and real-time data to generate actionable insights. Integration with existing systems, such as Enterprise resource planning (ERP) platforms, ensures seamless feedback for decision-making. Regular validation and fine-tuning of AI models maintain their relevance as operational conditions evolve [131]. Ongoing monitoring and refinement of AI systems are essential to ensure continuous improvement. Real-time data from IoT sensors can enhance the adaptability of AI tools [132]. Periodic updates and evaluations allow AI systems to incorporate advancements and align with changing operational goals. AI can significantly improve decision-making accuracy by analysing large datasets far beyond human capacity, providing actionable insights that drive operational improvements, and reducing costs and downtime while improving efficiency [133, 134].

This subsection reflects findings from the review where deep learning and computational simulations were identified as emerging but underutilised methods (Fig. 7). Their limited adoption was attributed to technical complexity and data requirements (Figs. 12 and 13), directly informing the guide’s emphasis on AI integration as a structured, staged activity rather than an ad-hoc addition.

### Research & development (R&D

This area underscores the importance of continuous development to ensure decision-making tools remain relevant and effective beyond initial adoption. The progression typically moves from scoping new pilots, through testing in controlled settings, to full integration or transfer into operations.

Sustained improvement is essential for long-term success. Organisations should remain attentive to emerging technologies by monitoring industry trends and conducting small-scale pilot projects before wider deployment. This iterative approach allows processes and tools to be refined in response to new findings, keeping them competitive and adaptable to evolving standards. Regular reviews provide a mechanism for incorporating advances and embedding lessons learned, thereby maintaining performance and ensuring that decision-making systems continue to deliver value over time.

The need for iterative R&D directly stems from the review’s observation that certain tools (e.g., VSM, Direct Comparison) have narrow applicability (Fig. 13) and require adaptation or combination with other methods to achieve broader impact. This supports the guide’s emphasis on pilot testing and incremental refinement as a way to operationalise hybrid strategies identified in the synthesis of findings.

### Leadership support and technology scalability

Ongoing management and leadership involvement are essential for ensuring that the integration of decision-making tools aligns with overarching business goals. Support from leadership teams can drive successful adoption by providing the necessary resources, setting priorities, and ensuring cross-departmental alignment. Without leadership backing, the implementation process may lack direction or stall altogether [135].

One of the key challenges businesses face is the evolving nature of technology. Companies must remain adaptable by integrating new decision-making tools and upgrading the infrastructure around them. Scalability should be a priority from the outset, allowing businesses to adjust their decision-making tools to accommodate future growth or changes in operational demands. As the tools evolve, businesses should stay open to upgrading their technology, ensuring that they continue to provide value over time.

### Summary and implications

The synthesis shows that effective integration of decision-making tools in manufacturing depends on clear data foundations, organisational readiness, and fit-for-purpose analytics that remain aligned with operational goals. The guide addresses gaps identified earlier by moving beyond isolated use cases and one-off implementations to a staged, system-level pathway for adoption. It provides a structured approach to integrating decision-making tools into manufacturing, addressing challenges in data handling, scalability and workforce readiness. By aligning with Industry 4.0 and sustainability goals, it ensures tools are adaptable to evolving needs while improving operational efficiency. Businesses can use this phased approach to implement tools effectively, from pre-implementation planning to post-implementation monitoring, enabling continuous improvement and long-term competitiveness.

## Conclusion

This study presents a comprehensive analysis of decision-making tools in manufacturing systems, offering both a thematic overview and an in-depth discussion of their practical applications across various operational levels. Through the review of selected research papers, several key insights emerged that hold important implications for both practice and future research.

The thematic analysis identified MCDA as the most commonly utilised tool, especially due to its ability to evaluate multiple conflicting criteria in complex decision-making environments. The increased adoption of sustainability-focused methods, such as LCA, highlights the growing importance of environmental considerations in the manufacturing sector. However, the limited application of LCA across studies indicates that there is potential for greater integration of sustainability metrics into decision-making frameworks. Tools like direct comparison and VSM were also prominent, emphasising the balance between simplicity and comprehensive analysis when selecting tools that fit specific manufacturing needs.

An important takeaway is the varying degrees to which these tools can be applied across different operational levels. While methods like MCDA and direct comparison provide valuable insights at the process level, they also possess the potential to inform higher-level decisions at the facility level. This adaptability, however, depends on the complexity of the tool. Advanced methods like deep learning and computational simulations, while powerful for process-level analysis, face challenges in scalability due to data requirements and computational complexity. These findings reinforce the need for both adaptable and specialised tools to address different decision-making needs across operational scales.

This research contributes to the field of manufacturing systems by outlining the current landscape of decision-making tools, shedding light on trends such as the growing reliance on data-driven methods and highlighting the continuous effort to integrate sustainability into manufacturing processes. Additionally, the guidelines provided outline essential steps for businesses looking to adopt decision-making tools in an increasingly digitised and data-driven environment, focusing on areas like AI integration, data management and scalability, which are critical in an increasingly digitised and interconnected manufacturing environment.

For academia, this study provides a foundation for future research into the development of more adaptable and multi-functional decision-making tools. Researchers can build on this work to explore synergies between existing tools, investigate their integration with real-time data and AI, and design frameworks that consider environmental, economic and social dimensions. Practitioners can leverage the provided guide to effectively implement decision-making tools, addressing challenges such as data standardisation, user-friendliness and scalability. By aligning operational strategies with technological advancements and sustainability goals, particularly within the context of Industry 4.0, practitioners gain actionable insights to enhance both competitiveness and regulatory compliance.

This work directly supports several SDGs by promoting sustainability and efficiency in manufacturing processes. Tools like LCA and sustainability metrics contribute to Goal 12 (Responsible Consumption and Production) by fostering resource efficiency and minimising environmental impacts. The integration of AI and data-driven methods aligns with Goal 9 (Industry, Innovation, and Infrastructure) by advancing innovation and enhancing manufacturing technologies. Furthermore, the emphasis on scalability, user-friendliness, and workforce readiness addresses Goal 8 (Decent Work and Economic Growth) by ensuring inclusive adoption of advanced tools across diverse manufacturing environments.

Future work should link process-centric decision tools to supply-chain decision layers through shared data models, supplier-facing indicators, and scenario-capable analytics. Priority areas include tighter integration with real-time data and AI to improve responsiveness, development of multi-dimensional frameworks that combine environmental, economic, and social metrics across sectors, and simplification of methods to improve user-friendliness and scalability. Data standardisation remains essential for reliable application, particularly for data-intensive tools. To extend impact beyond the plant, methods should propagate process metrics upstream and downstream and support supplier and route selection. Establishing interoperable interfaces and chain-of-custody data, and defining KPIs that connect process performance to supply-chain outcomes, will enable optimisation at the level of networks rather than isolated sites. These steps position decision-making tools to support sustainable and resilient manufacturing ecosystems.

## Appendix

See Table 6.

**Table 6:** Summary of the explored literature

	Source	Date	Aim	Method	Aspect
1	[42]	2024	Compares the sustainability of two metal AM methods	A TOPSIS-based analysis was used with multiple indicators	Environmental and economic
2	[43]	2022	Develops a decision support system to select a 3D printing technology	An evaluation criteria list using a posteriori-based MCDA approach	Resource performance capabilities and manufacturing requirements
3	[44]	2023	Systematically analyses CO_2_ capture technologies for the cement industry	Techno-economic analysis was conducted to recommend optimal technologies using MCDA	Technical, economic, and environmental impacts
4	[45]	2020	Proposes a method to select the best manufacturing technology based on several criteria	Uses a TOPSIS-based MCDA approach	Cost, performance, and resource availability
5	[46]	2021	Compares EDM and laser processes for creating cooling holes in gas turbines	Multiple MCDA methods were applied	Energy consumption, material use and process efficiency
6	[47]	2016	Compares selective laser sintering with other AM processes	Direct comparison of time–cost and quality analysis of processes	Focuses on time, cost, and surface quality
7	[48]	2022	Develops a multi-criteria decision support system for machining process selection	Uses PROMETHEE multi-criteria decision-making approach	Economic and productivity criteria, with a focus on sustainability
8	[49]	2017	Compares traditional and AM methods in the aerospace industry	Applies AHP, SPA, and VDI 2225 decision-making methods	Production characteristics including raw materials, energy, time and cost
9	[50]	2015	Proposes a new metric (OEEE) to evaluate environmental effectiveness in manufacturing	Uses the OEEE metric to compare environmental impacts	Environmental sustainability and process efficiency
10	[51]	2021	Compares finish machining and mass finishing for AM competitiveness	Direct comparison of surface quality	Surface roughness and post-processing effectiveness
11	[52]	2024	Selection of a suitable AM process using a three-way decision-making model	TOPSIS, MOORA and VIKOR to rank AM processes	Mechanical properties of the product
12	[53]	2016	Tailoring soy protein film properties by selecting manufacturing processes	LCA to compare environmental impacts of wet and dry processes	Environmental impacts and performance of the processes
13	[54]	2020	Develops a process selection methodology based on economic and environmental performance	Fuzzy logic and LCA	Manufacturing costs and environmental trade-offs
14	[55]	2019	Process and resource selection methodology in AM design	AHP-based decision-making framework	Economic analysis and performance efficiency
15	[56]	2023	Examines additive and subtractive manufacturing	LCA and LCC	Sustainability and cost-effectiveness
16	[57]	2021	Compare hybrid additive-subtractive manufacturing	LCEA model using primary energy demand as the evaluation metric	Energy demand and utilisation efficiency
17	[58]	2015	Evaluate energy performance and provide improvement guidelines	Energy performance benchmarking	Energy performance
18	[59]	2021	Evaluate renewable diesel production	TOPSIS analysis	Environmental impact and economic sustainability
19	[60]	2023	Develops a hybrid decision-making framework for AM process selection	CRITIC and EDAS-based multi-criteria decision-making approach	Surface roughness and dimensional accuracy
20	[61]	2021	Compares additive and subtractive rapid prototyping techniques	AHP analysis	Surface roughness, cost and processing time
21	[62]	2022	Develops a context-based decision support system for energy-efficient manufacturing	Monitoring and decision-support framework for energy efficiency	Impact on energy performance
22	[63]	2014	Compares fuzzy linguistic approaches for material selection in custom prosthetics manufacturing	Fuzzy AHP decision-making method	Material selection, custom fit and manufacturing efficiency
23	[64]	2016	Compares the environmental impacts of additive versus traditional manufacturing technologies	Environmental impact comparison using LCA tools	Relative environmental impact of processes
24	[65]	2020	Presents an MCDA approach for AM process selection	MCDM approach based on process performance assessment	Process performance and efficiency
25	[66]	2020	Develops a decision support system for selecting AM processes based on benchmarking	Benchmarking and decision-support system	Performance benchmarking and process selection in AM
26	[67]	2022	Develops a multi-criteria decision-making method for AM process selection	MCDA tool with scenario analysis	Evaluates process performance across different manufacturing scenarios
27	[68]	2023	Presents fuzzy-based models for the classification of machining and production data	Fuzzy logic-based classification method	Data ambiguity reduction in machining and production processes
28	[69]	2023	Develops a framework for selecting materials and manufacturing technologies	SAW, TOPSIS and PROMETHEE were used	Material and manufacturing process performance
29	[70]	2021	Assesses the utility of owning versus outsourcing 3D printing jobs	Estimated utilisation factor and fuzzy sets	Cost, tardiness, energy and waste
30	[71]	2019	Develops an online decision-support tool for selecting technology in biopharmaceutical manufacturing	Decision-support tool based on an aggregated score from a set of indicators	Economic, environmental, social and quality metrics
31	[72]	2019	Assess environmental performance in cooling tower operations	A scenario-based approach using best-case and worst-case analyses based on a set of indicators	Efficiency, environmental, energy and economic performance
32	[73]	2023	Develops an advanced process for Lean manufacturing approach	Developed an integrated MCDA-VSM model	Environmental sustainability and process efficiency indicators
33	[74]	2020	Conducts an environmental analysis of selective laser melting in AM	LCA for evaluating environmental impacts	Environmental performance
34	[75]	2022	Proposes a system model for robust tablet manufacturing process selection	Computational simulation for process modelling	Process efficiency and product quality
35	[76]	2020	Selects the best green manufacturing process for oil production	AHP method is used to perform an evaluation of pairwise comparisons	Environmental sustainability, economic performance and availability
36	[77]	2023	Develops a decision support system for selecting robust manufacturing processes in food processing	MCDA approach	Process reliability and food safety
37	[78]	2024	Evaluates the environmental footprint of additive and subtractive manufacturing processes	LCA and process benchmarking	Environmental impact and resource efficiency
